# Direct Ink Writing 3D Printing for High‐Performance Electrochemical Energy Storage Devices: A Minireview

**DOI:** 10.1002/advs.202303716

**Published:** 2023-09-22

**Authors:** Li Zeng, Shangwen Ling, Dayue Du, Hanna He, Xiaolong Li, Chuhong Zhang

**Affiliations:** ^1^ State Key Laboratory of Polymer Materials Engineering Polymer Research Institute Sichuan University Chengdu 610065 P. R. China

**Keywords:** 3D printing, direct ink writings, electrochemical energy storage devices, batteries, supercapacitors

## Abstract

Despite tremendous efforts that have been dedicated to high‐performance electrochemical energy storage devices (EESDs), traditional electrode fabrication processes still face the daunting challenge of limited energy/power density or compromised mechanical compliance. 3D thick electrodes can maximize the utilization of *z*‐axis space to enhance the energy density of EESDs but still suffer from limitations in terms of poor mechanical stability and sluggish electron/ion transport. Direct ink writing (DIW), an eminent branch of 3D printing technology, has gained popularity in the manufacture of 3D electrodes with intricately designed architectures and rationally regulated porosity, promoting a triple boost in areal mass loading, ion diffusion kinetics, and mechanical flexibility. This focus review highlights the fundamentals of printable inks and typical configurations of 3D‐printed devices. In particular, preparation strategies for high‐performance and multifunctional 3D‐printed EESDs are systemically discussed and classified according to performance evaluation metrics such as high areal energy density, high power density, high volumetric energy density, and mechanical flexibility. Challenges and prospects for the fabrication of high‐performance 3D‐printed EESDs are outlined, aiming to provide valuable insights into this thriving field.

## Graphical abstract

This review comprehensively discusses and categorizes the efficient optimization strategies for achieving high‐performance and multifunctional 3D‐printed EESDs, based on performance evaluation metrics such as high areal energy density, high power density, high volumetric energy density, and superior mechanical flexibility. Challenges and prospects for the fabrication of high‐performance 3D‐printed EESDs are outlined, aiming to provide valuable insights into this thriving field.

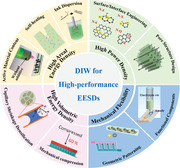

## 1 Introduction

Electrochemical energy storage devices (EESDs) have been an integral part of modern human life with the widespread use of smartphones and electric vehicles due to their exceptional properties, including high energy density, high power density, and long lifespan.^[^ ^1^, ^2^ ^]^ With the rapid development of the Internet of Things (IoT) and wearable devices, the demand for electronic devices to be miniaturized, intelligent, and multifunctional is constantly increasing.^[^ ^3^, ^4^ ^]^ EESDs not only need to meet the requirements of miniaturization, flexibility, lightweight, and multifunctionality, but also need to have high energy/power density, high safety, and stable cycling performance.^[^ ^5^, ^6^ ^]^ However, traditional EESDs are hindered by limited mass loading, inadequate ion diffusion kinetics, and inferior mechanical robustness, resulting in insufficient energy storage efficiency and electrode/device‐level flexibility.^[^ ^7^, ^8^, ^9^ ^]^

Over the past few decades, research on high‐performance electrode materials has been given top priority.^[^ ^10^, ^11^, ^12^, ^13^ ^]^ Despite significant innovations and successes, the manufacture of tunable electrode propulsion structures remains a slow process.^[^ ^14^, ^15^ ^]^ Traditional manufacturing technologies, such as the 2D slurry‐coating method, have difficulty achieving customized designs with a diverse range of electrode structures, and face challenges such as low mass loading, complex processes, and high preparation costs. The thick electrode with a 3D structure can effectively utilize the *z*‐axis space to achieve higher mass loading of active materials, ultimately maximizing the energy density of the device. However, the sluggish electron/ion transfer and mechanical instability of the electrode as the thickness increases severely hinder their practical applications. In this context, 3D electrodes with periodic or aligned pore structures featuring high mass loading and fast ion transport in a limited footprint, which is beneficial for fast charge–discharge and enhanced energy density, are highly required for advanced miniaturized and customized EESDs.^[^ ^16^, ^17^ ^]^

Currently, 3D printing technology, also known as additive manufacturing (AM), utilizes layer‐by‐layer deposition and computer‐aided design (CAD) drawing to produce highly integrated freeform construction and controllable 3D structural prototyping economically and efficiently. In particular, 3D‐printed EESDs offer several advantages, including the ability to fabricate electrodes with arbitrary geometry and increased mass loading, customizable composition and physical properties of the fabricated devices, and reduced manufacturing costs and material wastage.^[^ ^18^ ^]^ These merits make 3D printing technology attractive for the development of high‐performance 3D EESDs. There are four representative technologies that are available for the fabrication of EESD modules so far, including direct ink writing (DIW), stereo lithography apparatus (SLA),^[^ ^19^, ^20^ ^]^ selected laser sintering (SLS),^[^ ^21^, ^22^ ^]^ and fused deposition modeling (FDM).^[^ ^23^, ^24^ ^]^ The above four technologies differentiate themselves based on the technical processes employed to prepare EESDs, including material extrusion for DIW or FDM, powder bed fusion for SLS, and vat photopolymerization for SLA, thereby exhibiting variations in printable materials, resolution, cost, and design versatility. Each technology sets unique requirements for suitable printable materials, exemplified by the necessity of UV‐curable photopolymer resins in SLA, metal powders in SLS, and thermoplastic materials in FDM. The efficient dispersion of active materials in the ink and complex post‐processing of the printed electrodes pose additional challenges for the fabrication of high‐performance EESD modules using SLA, SLS, and FDM, not to mention the demanding and complex manufacturing conditions that are always required (such as UV lasers and high temperatures).

Direct ink writing (DIW) is currently the most widely used 3D printing technology due to its versatility in material selection, high throughput capabilities with multi‐nozzle systems, and facile printing mechanism for all degrees of freedom with customized free‐standing electrodes.^[^ ^25^ ^]^ In fact, it has been reported that DIW constitutes ≈70% of 3D‐printed EESDs, and this percentage is expected to rise as DIW becomes more extensively utilized. DIW technology adopts a specialized printer to extrude a high‐viscosity slurry through a nozzle, which can be easily modified by incorporating external stimuli such as magnetic fields, electromagnetic waves, acoustic waves, and heat sources. By stacking the printed layers along the *z*‐axis, complex spatial structures with controlled microstructures and high mass loading of active materials can be rapidly prototyped, delivering a high energy output in a limited footprint. The versatility of DIW has been the catalyst for extensive research into the advancement of energy storage systems such as lithium‐ion batteries (LIBs),^[^ ^26^ ^]^ lithium‐sulfur batteries (LSBs),^[^ ^27^ ^]^ zinc ion batteries (ZIBs),^[^ ^28^ ^]^ and supercapacitors (SCs).^[^ ^29^ ^]^ For example, in 2013, Lewis et al.^[^ ^30^ ^]^ reported for the first time a well‐designed 3D‐printed architecture with organized macropores prepared using DIW method. The 3D scaffold with increased surface area facilitated full penetration of the electrolyte, achieving high capacity and long‐term cycling stability of LIBs. Later in 2016, a self‐standing 3D graphene‐based microlattice was fabricated by DIW 3D printing and served as the SCs electrode with exceptional capacitive retention and power density.^[^ ^31^ ^]^ To date, although DIW 3D printing has made numerous remarkable advances in EESDs, there are still challenges that need to be overcome in order to achieve higher performance and more functionality. For example, the energy output of thick 3D‐printed electrodes is hampered by interlayer resistance resulting from layer‐by‐layer stacking manner and insufficient utilization of active materials. In addition, ion diffusion kinetics within 3D electrodes is less than optimal due to the tortuous ion diffusion path. Most 3D electrodes prepared by DIW are loose and porous after post‐treatment (i.e., solvent removal, heat treatment), which also results in a generally low volumetric energy density. Furthermore, due to the unavoidable influence of printed materials, the mechanical strength of the printed electrode needs to be further enhanced.

**Figure 1:**
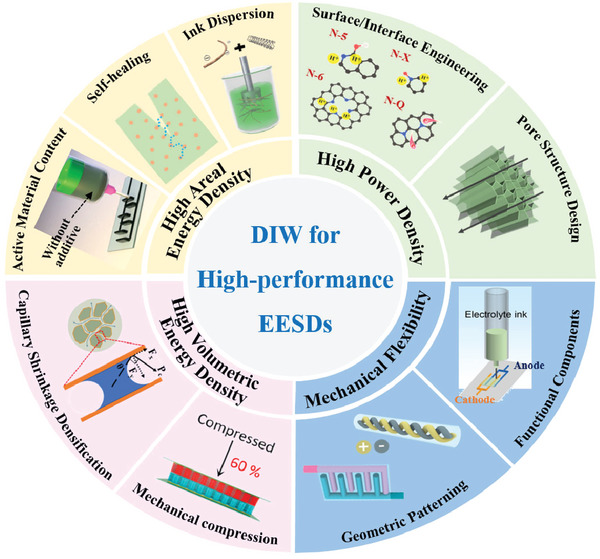
Overview of high‐performance EESDs using DIW technology.

Therefore, this review aims to highlight the pivotal role of DIW 3D printing in fabricating high‐performance EESDs. Firstly, the basic properties of printable inks and the typical DIW 3D printed configurations of EESDs are introduced. We then systematically discussed the valuable strategies for achieving high performance and multifunctional EESDs by classifying the critical performance characteristics, including areal energy density, power density, volumetric energy density, mechanical flexibility, etc. (**Figure** **1**). Finally, conclusions and prospects for the development of printed EESDs are outlined, highlighting the current challenges and future perspectives for DIW 3D printing, which could provide an important avenue for future research.

## 2 The Basic Properties of Printable Inks

### 2.1 Rheological Requirements of DIW Inks

The apparent shear‐thinning behavior and ideal modulus of fluid DIW inks are crucial characteristics that ensure excellent printability and precise shape retention of the self‐supporting layers produced through DIW printing.

**Figure 2:**
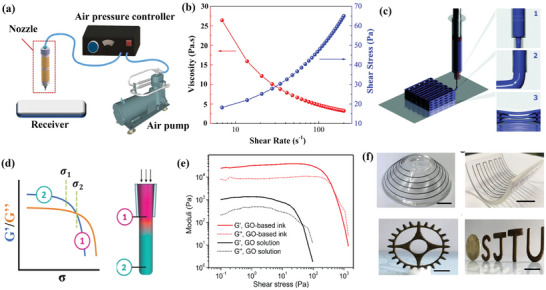
a) The DIW 3D printing process. Reproduced with permission.^[^ ^32^ ^]^ Copyright 2016, Springer Nature Ltd. b) Log‐log plots of viscosity versus shear rate with a typical shear‐thinning behavior. Reproduced with permission.^[^ ^40^ ^]^ Copyright 2019, Elsevier. c) Schematic showing the ideal configuration process of customized self‐standing 3D scaffold. Reproduced with permission.^[^ ^45^ ^]^ Copyright 2021, Royal Society of Chemistry. d) *G*′ and *G*″ versus shear rate of a printable ink and the corresponding pressure‐induced flow states. Reproduced with permission.^[^ ^25^ ^]^ Copyright 2020, Wiley‐VCH. e) Log‐log plots of *G*′ and *G*″ versus shear stress with/without the modulus regulation of GO‐based inks and solutions, and f) the complicated 3D structures constructed by the modified GO‐based ink. Reproduced with permission.^[^ ^48^ ^]^ Copyright 2018, American Chemical Society.

In general, in an ideal DIW 3D printing process (**Figure** **2**a), the inks are typically extruded through a syringe barrel and nozzle to form filaments under external pressure.^[^ ^32^ ^]^ The resulting 3D structure is constructed layer‐by‐layer by depositing the filaments onto a lower receiver in accordance with a pre‐determined CAD model.^[^ ^33^, ^34^, ^35^ ^]^ To ensure printability, it is crucial that the inks flow smoothly without any particle jamming, which could lead to clogging of the print nozzle and cause discontinuity in the printing process.^[^ ^36^, ^37^ ^]^ From a rheological perspective, the shear deformation capability is a critical indicator that influences the properties of the ink. Conventional fluids can be categorized based on their viscosity behavior into two types: Newtonian (constant viscosity with changing shear rate) and non‐Newtonian (varying viscosity with changing shear rate) fluids.^[^ ^38^ ^]^ Non‐Newtonian fluids can be further subdivided into shear‐thinning and shear‐thickening fluids. The viscosity of shear‐thickening fluids increases with increasing shear stress, which can cause awful blocking issues during the extrusion process from the nozzle.^[^ ^39^ ^]^ In contrast, the shear‐thinning fluid has a decreasing viscosity as the shear rate increases (Figure 2b), enabling the ink to flow smoothly through the nozzle even at low extrusion stress, thus improving printability.^[^ ^40^ ^]^ Typically, shear‐thinning DIW ink exhibits a viscosity range of 10^−2^ to 10^4^ Pa·s at a shear rate of ≈0.1 s^−1^.^[^ ^41^, ^42^ ^]^ This viscosity range is well‐suited for various printing applications, providing excellent flow properties that enable the ink to extrude smoothly through the nozzle while maintaining its shape and consistency on the substrate.

Theoretical Herschel‐Bulkley model reveals the relationship between yield stress and shear stress of fluids, where the model is given by,

(1) $$\tau =\tau_{y}+K{\dot{\gamma }}^{n}$$

Where τ is the shear stress, τ_y_ is the yield shear stress, K is the viscosity index, $\dot{\gamma }$ is the shear rate, and n is the fluid flow index. When the imposed shear stress exceeds the yield stress, gelatinous inks could transform to a fluid‐like state and exhibit a shear‐thinning behavior, characterized by an exponent n ranging from 0 to 1.^[^ ^43^ ^]^ This means that the viscosity of the ink decreases as the shear rate increases, allowing for smooth and consistent flow during printing. It is easy to calculate the exact values of n and K by plotting the shear stresses and corresponding shear rates in a log‐log formation. These values filter out the ideal shear‐thinning inks and guide the optimization of DIW ink compositions.

Printable inks are also required to possess a sufficiently high storage modulus to ensure excellent self‐standing properties, which guarantee dimensional stability and prevent undesired flow of the constructed 3D structures.^[^ ^44^ ^]^ As illustrated in Figure 2c, once the printing filament exits the nozzle, it deposits on top of the previously printed layers or the bottom substrate, and the ink is no longer subjected to shear stress.^[^ ^45^ ^]^ Numerous non‐Newtonian fluids are viscoelastic in nature, displaying a combination of liquid‐like viscous behavior and solid‐like elastic behavior. The storage modulus (*G*') and loss modulus (*G*“) indicate the elasticity and viscosity of the ink, respectively, while the ratio of *G*' and *G*” corresponds to the shape retention capability.^[^ ^46^ ^]^ As shown in Figure 2d, ink fluids transition into a liquid state and flow through a narrow nozzle as a result of the stretched and deformed bonds between atoms or molecules under the influence of shear stress (region 1). Subsequently, when the printed filaments deposit on the substrate, the ink fluids revert to a solid‐like state as the shear stress is relieved. In this state, the 3D structure of the printed object can be maintained due to the immediate recovery of the elastic part of the total deformation, while the viscous part gradually recovers, eventually reaching an equilibrated viscoelastic state (region 2).^[^ ^25^ ^]^ The yield stress is defined as the shear stress required to initiate the transformation from a solid to a liquid state. In particular, to prevent structural collapse after DIW printing, it is crucial to have a higher *G*’ than *G*’’ at low strain stress. This ensures that the material can withstand the shear stress during printing without undergoing significant deformation.^[^ ^47^ ^]^ As exhibited in Figure 2e,f, the introduction of a mild cross‐linker into the GO‐based ink could effectively improve the *G*′ and *G*″ by an order of magnitude compared to the pure GO solution, ensuring the strong dimensional stability of the printed rigid filament.^[^ ^48^ ^]^

### 2.2 Rheological Regulation

**Figure 3:**
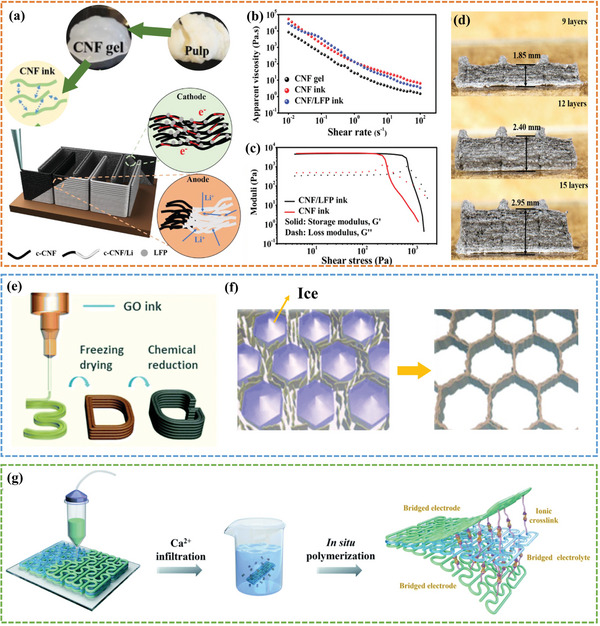
a) Schematic representation of 3D printed CNF‐based scaffold of LFP cathode and metal Li anode. b) Apparent viscosity as a function of shear rate and c) *G*’ and *G*’’ as a function of shear stress for inks. d) The thickness increase linearly with stacked printed layers. Reproduced with permission.^[^ ^55^ ^]^ Copyright 2019, Wiley‐VCH. e) 3D‐printed graphene architecture prepared by GO ink; f) Formation of the 3D GO‐based architecture with the ice‐templating technique. Reproduced with permission.^[^ ^64^ ^]^ Copyright 2017, Royal Society of Chemistry. g) The illustration of the Ca^2+^ gelation process targeting the preparation of MXene ink and the 3D printing process. Reproduced with permission.^[^ ^66^ ^]^ Copyright 2022, Royal Society of Chemistry.

Viscosity and thixotropy are crucial factors in the preparation of printable inks, hence the selection of appropriate rheological modifiers is essential to the DIW manufacturing process. Polymers are the most commonly used substances to regulate the rheological behavior of DIW inks. There are various types of polymer rheological modifiers that can be employed, including cellulose,^[^ ^49^ ^]^ sodium alginate (SA),^[^ ^50^ ^]^ sodium carboxymethyl cellulose (CMC),^[^ ^51^ ^]^ polyvinyl alcohol (PVA),^[^ ^52^ ^]^ carbomer,^[^ ^53^ ^]^ hyaluronic acid (HA),^[^ ^54^ ^]^ polytetrafluoroethylene (PVDF)^[^ ^46^ ^]^ etc. Generally, the rheological regulator chosen for a particular ink formulation can have a significant impact on its viscosity, thixotropy, and printability, thus requiring meticulous selection to ensure the desired rheological behavior and optimal performance of the final printed product. For example, Zhu et al.^[^ ^55^ ^]^ successfully prepared high‐performance lithium metal micro‐batteries enabled by adopting nanocellulose as the rheological regulators, the schematic representation of printing process is shown in **Figure** **3**a. The incorporation of shear‐thinning nanocellulose gel ensured the printability of electrode ink, while also providing a stable scaffold for the Li metal (Figure 3b,c). Meanwhile, the good dimensional stability allowed the thickness to increase linearly as the printing layers were stacked (Figure 3d). Later, Zhang et al.^[^ ^56^ ^]^ formulated a range of DIW inks with cellulose as the regulating agent, incorporating gradient Ag nanoparticles as zincophilic materials. All inks, including those with Ag concentrations of 0 wt%, 5 wt%, and 10 wt%, exhibited high storage modulus and excellent shear‐thinning properties, which promoted the dimensional stability of the 3D‐printed scaffold. In addition to the direct use of viscoelastic polymers as rheological modifiers, Lu et al.^[^ ^57^ ^]^ creatively proposed a strategy to prepare printable inks by harnessing acrylamide monomers and incorporating EGaIn microdroplets to induce polymerization and chain entanglement. The resultant hydrogel, comprised of polyacrylamide‐hemicellulose/EGaIn microdroplets, showcases a dual covalent hydrogen bonding system that imparts self‐healing capabilities and shear‐thinning properties. As a result, this versatile hydrogel not only serves as an extrudable ink but also functions as an electrically/ionically conductive microporous matrix and anode host for Zn‐ion batteries.

In addition, 2D sheet materials with abundant hydroxyl and carboxyl groups, such as graphene oxide (GO),^[^ ^31^, ^58^, ^59^ ^]^ MXene,^[^ ^60^, ^61^, ^62^ ^]^ have emerged as promising electroactive candidates for modifying rheological behavior and formulating printable inks for a broad range of applications. For example, Zhang et al.^[^ ^63^ ^]^ adopted a simple strategy to prepare a high‐concentrated GO ink of 200 mg mL^−1^ in NaOH solution that meets the stringent rheological requirements for 3D printing of supercapacitors. Advantageously, the ink formulation is simple and scalable, and the porosity of the printed 2D sheet materials can be easily regulated with a further freeze‐drying process. Li et al.^[^ ^64^ ^]^ prepared a honeycomb porous GO structure transferred from the original macroscopic structure by combining DIW printing with freeze‐drying under different temperatures (Figure 3e). The GO sheets were forced to align along the boundaries of the ice crystals, gradually forming cellular networks through cross‐linking of adjacent GO sheets via π‐π interactions (Figure 3f). Fabricated with optimized porosity and high mechanical flexibility, the supercapacitors retained 97% of initial capacitance after 200 cycles at a bending angle of 60°.

Physical and chemical cross‐linking can serve as highly efficacious approaches for augmenting the interconnectivity of 2D sheet materials within printable inks even with ultra‐low concentrations. Gao et al.^[^ ^65^ ^]^ converted aqueous GO solutions (20 mg mL^−1^) into printable gel inks by adding trace amounts of Ca^2+^ ions. The resulting GO‐based hydrogel ink gave shear‐thinning rheological behaviors with high apparent viscosity. Guan et al.^[^ ^66^ ^]^ successfully prepared an MXene‐based ink for DIW 3D printing using a rapid gelation process with a trace amount of Ca ^2+^ ions. As shown in Figure 3g, Ca^2+^ ions could be readily adsorbed onto the negatively charged MXene surface due to electrostatic attraction, ensuring the printability of the DIW process. Other multivalent ions such as Mg^2+^,^[^ ^67^ ^]^ Al^3+^,^[^ ^68^ ^]^ and Fe^2+[^ ^69^ ^]^ can also be used as gelatinizing agents for DIW ink systems.

Apart from the aforementioned strategies, inorganic nanoparticles have also been utilized to regulate the rheological properties. Worsley et al.^[^ ^70^ ^]^ used silica to increase the viscosity of GO‐based ink for 3D‐printed supercapacitors. Incorporation of silica fillers yielded a significant increase in both the elastic modulus *G*’ and yield stress of the ink, by more than one order of magnitude. This suggests that the printed structures possess superior dimensional stability compared to those printed without silica. Despite the benefits of incorporating silica fillers into ink formulations, the post‐processing required for silica‐etching can complicate the manufacturing process. Additionally, dielectric silica particles can negatively impact the electrochemical performance of 3D‐printed electrodes, further limiting the use of silica fillers in ink formulations.

Despite variations in the mechanisms that govern the regulation of rheological behavior by diverse rheological modifiers, they all adhere to a fundamental principle of establishing and collapsing stable structures in the ink. In the case of polymers, the entanglement or cross‐linking of polymer chains enables the ink to fulfill the modulus requirements, while the shearing forces cause chain orientation or disruption of the cross‐linked network, resulting in shear thinning properties of the ink. For 2D sheet materials, they form condensed and compressed liquid crystal domains at high concentrations or cellular structures under the effect of cross‐linking agents to promote solid phase behavior and shear thinning results from the breakdown of these structures. For surface‐functionalized inorganic nanoparticles (such as hydrophilic fumed silica), interactions like hydrogen bonding play a role in the formation of an aggregation network, which increases the viscosity of the inks. However, when an external shear force is applied, this aggregation network collapses, leading to a reduction in viscosity.

## 3 Typical DIW 3D‐Printed Configurations of EESDs

**Figure 4:**
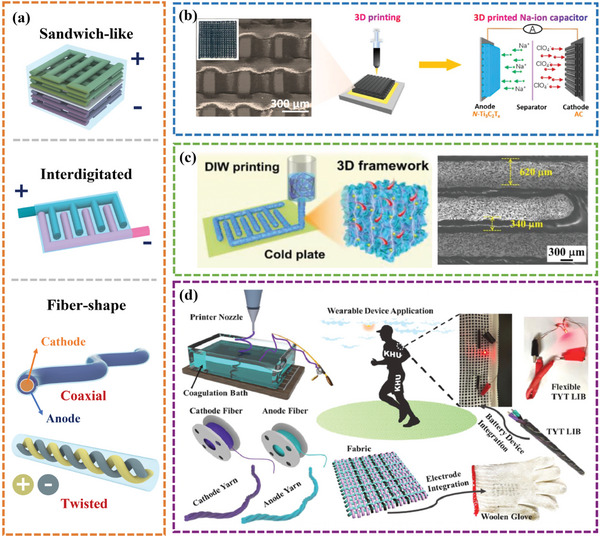
a) The main macrostructure of 3D printed electrodes. b) Schematic diagram showing the preparation process of 3D‐printed woodpile‐like electrodes. Reproduced with permission.^[^ ^75^ ^]^ Copyright 2020, American Chemical Society. c) Schematic illustration of the preparation of the DIW 3D‐printed interdigital electrodes. Reproduced with permission.^[^ ^79^ ^]^ Copyright 2023, Wiley‐VCH. d) Schematic illustration of the fabrication process of flexible fiber LIBs. Reproduced with permission.^[^ ^82^ ^]^ Copyright 2022, Elsevier.

3D electrodes, prepared using the DIW method, can be assembled into uniquely shaped devices to satisfy the requirements of various EESDs. The electrochemical performance of EESDs is greatly influenced by the different 3D structures, which can be regulated using pre‐set customized 3D printing models. The primary electrode structures include woodpile‐like, interdigitated, and fibers (**Figure** **4**a). The properties, advantages, and typical examples are discussed in this section.

### 3.1 Sandwich Configuration

The sandwich configuration, a classic prototype for cost‐effective production of EESDs, is widely used in coin‐type and gel‐type systems where two analog 3D electrodes are separated on either side of the separator.^[^ ^71^ ^]^ Generally, a woodpile‐like electrode is one of the most common 3D electrodes for sandwich configuration, which is primarily a macro aerogel electrode with an orthogonal lattice multilayer architecture. Multiple lattice layers are stacked in the *z*‐axis direction to obtain a 3D‐printed woodpile structure, leaving ordered pores and a high specific surface area to facilitate efficient ion diffusion and electrolyte penetration, thus achieving high energy/power performance.^[^ ^72^ ^]^ The porous structure of the woodpile‐like electrode offers several benefits. Firstly, it helps to minimize the local current density and optimize the utilization of active materials during the charge–discharge process. Additionally, this structure can enhance the mechanical properties of the electrode by reducing the mechanical stress within the electrode. Such a reduction is facilitated by the porous nature of the electrode, which enables a more even distribution of mechanical stress, thereby promoting heightened durability and prolonged lifespan of electrodes.^[^ ^73^, ^74^ ^]^ As shown in Figure 4b, Sun et al.^[^ ^75^ ^]^ demonstrated a 3D‐printed sodium‐ion hybrid capacitor (SIC) employing a woodpile‐like nitrogen‐doped MXene (N‐Ti_3_C_2_T_x_) anode and a woodpile‐like activated carbon cathode. Benefiting from the large surface area and efficient ion diffusion, the 3D‐printed SICs harvested an areal energy density of 1.18 mWh cm^–2^ and a power density of 40.15 mW cm^–2^, respectively.

The high porosity and regular network architecture of the 3D‐printed woodpile‐like aerogel electrode in sandwich configuration enables exceptional mechanical strength and satisfactory ion diffusion kinetics. However, the volumetric energy density of advanced EESDs is significantly diminished by the presence of excessive mesopores and macropores (a few microns to tens of microns in diameter) within the 3D‐printed porous aerogel electrodes, which are flooded with electrolyte but with little contribution to the mass‐specific capacity.

### 3.2 Interdigitated Configuration

The interdigitated configuration, as a conventional yet versatile structural design, has gained widespread popularity in the realm of micro‐sized and stretchable EESDs, engineered to seamlessly integrate with wearable technologies.^[^ ^76^, ^77^ ^]^ Through the utilization of a “finger” or “comb” electrode structure, the positive and negative electrodes are intricately intertwined, resulting in an expanded contact area and a shorter ion transfer distance. This design not only serves to diminish electronic resistance but also optimizes kinetics, culminating in an unparalleled level of performance. Yang et al.^[^ ^78^ ^]^ created a quasi‐solid asymmetric micro‐supercapacitor using 3D‐printed G‐VNQDs/GO and V_2_O_5_/GO inks in a three‐finger array shape for the first time. The micro‐supercapacitor showed outstanding structural integrity and an ultra‐high areal capacitance of 207.9 mF cm^−2^. Later, Liu et al.^[^ ^79^ ^]^ successfully prepared a 3D‐printed micro‐supercapacitor with interdigital electrodes. As demonstrated in Figure 4c, there is a small gap of 340 µm between two adjacent electrodes, which was favorable to fast ion/electron transportation. As a result, the printed all‐gel‐state micro‐supercapacitor exhibited a high areal capacitance of 889 mF cm^−2^ and superior rate performance with 80% capacitance retention under tenfold current density.

### 3.3 Fiber‐Shape Configuration

The 3D fiber electrode is renowned for its ability to readily conform to intricate deformations, including twisting and stretching. In contrast to conventional 2D and 3D electrodes, it boasts a compact form and exceptional knittability, rendering it a pivotal constituent of the flexible EESD landscape.^[^ ^80^, ^81^ ^]^ The prevalent layout for fiber‐shaped energy storage devices distinguished by the relative position of the two electrodes are categorized into two primary classes: coaxial and twisted structure. The coaxial structure encompasses an internal and external electrode that respectively functions as the positive and negative electrodes, with a central gel electrolyte that amalgamates the sandwiched structure. On the other hand, the twisted structure comprises two fiber electrodes that contain active materials, twisted together at a specific angle to form the positive and negative poles. Recently, Lee et al.^[^ ^82^ ^]^ reported a twisted configuration LIB using DIW technology with graphite and LiNi_0.6_Co_0.2_Mn_0.2_O_2_ working as the anode and cathode, respectively (Figure 4d). The fabricated twisted yarn‐type LIB device delivered a specific discharge capacity of 162 mAh g^−1^/2.5 mAh cm^−2^ at 0.1 C. Remarkably, the device demonstrated good weavability, which could be woven into a woolen glove and fully integrated into textile fabrics.

## 4 Preparation Strategies for High‐Performance and Multifunctional 3D‐Printed EESDs

### 4.1 High Areal Energy Density EESDs

The swift advancement of electric vehicles and intelligent electronic device technology has propelled the surge of interest in high‐areal energy density EESDs research. Through the utilization of layer‐by‐layer stacking and bottom‐up assembly process characteristics, 3D printing technology facilitates a substantial augmentation in areal mass loading, while concurrently preserving the outstanding mechanical properties of the printed electrodes and significantly enhancing the ion transport capability. Nevertheless, the energy output of thick 3D‐printed electrodes is impeded by the notorious interlayer resistance, which is a consequence of the layer‐by‐layer stacking process, and the suboptimal utilization rate of active materials. To overcome these challenges, researchers have endeavored to enhance DIW technology by eliminating interlayer resistance and augmenting active substance utilization.

#### 4.1.1 Uniform Ink Dispersion Improving the Utilization of Active Materials

**Figure 5:**
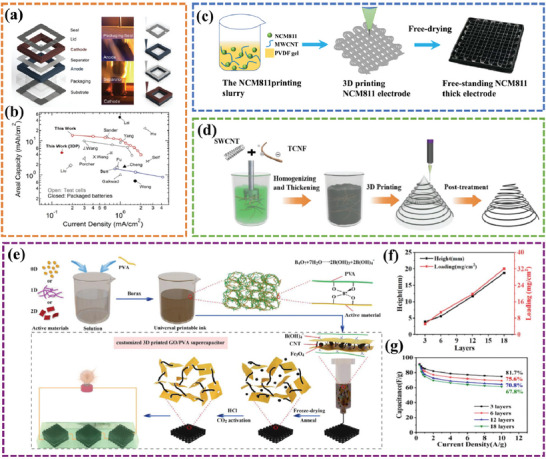
a) Schematic representation and b) the capacities under different current densities of fully 3D printed Li‐ion square cell battery. Reproduced with permission.^[^ ^83^ ^]^ Copyright 2013, Wiley‐VCH. c) an NCM 811/SWCNT/PVDF composite as 3D printable ink for LIB electrodes. Reproduced with permission.^[^ ^84^ ^]^ Copyright 2022, American Chemical Society. d) An overall schematic diagram illustrating the self‐healing ink and 3D printing process. Reproduced with permission.^[^ ^85^ ^]^ Copyright 2021, Wiley‐VCH. e) The schematic representation of the gelation‐induced ink‐enabled 3D printing process. f) The linearly‐increased mass loading and g) areal capacity with an excellent healing effect. Reproduced with permission.^[^ ^52^ ^]^ Copyright 2021, Elsevier.

The essential factors for achieving a continuous DIW process with an optimal ink are 1) proper rheology and clog‐resistant properties, 2) strong adhesion and wetting characteristics with the substrate, and 3) consistency and steadiness without any settling or re‐agglomeration for an extended duration. Typically, 3D‐printable inks consist of various elements such as active materials, binders, conductive additives, and other components. In general, in the absence of a dispersant, active material particles readily agglomerate as a result of their innate van der Waals forces under high ionic strength conditions, leading to undesirable material aggregation and diminished utilization. As a consequence, the attainment of ink homogeneity represents a pivotal factor in advancing energy storage capabilities and elevating the energy density of EESDs to unprecedented levels. Lewis et al.^[^ ^83^ ^]^ reported fully 3D‐printed LIBs consisting of thick and biphasic semi‐solid electrodes (**Figure** **5**a). A nonionic poly(vinylpyrrolidone) (PVP) dispersant was adopted to stably disperse the active nanoparticles (LTO and LFP) and improve their utilization efficiency. As a result, the fabricated fully 3D‐printed, and packaged LIBs achieved a high areal capacity of 4.45 mAh cm^−2^ at 0.14 mA cm^−2^ (Figure 5b). Tang et al.^[^ ^84^ ^]^ also reported an NCM 811/SWCNT/PVDF composite as 3D printable ink for LIB electrodes (Figure 5c). The composition of the prepared ink showed good rheological properties through batch dispersion, and the printed LIB electrode delivered a high areal capacity of up to 7.48 mAh cm^−2^ at 16.56 mA cm^−2^.

#### 4.1.2 Self‐Healing Strategy Eliminating Interlayer Resistance

The layer‐by‐layer strategy utilizing DIW technology presents a favorable avenue for achieving high mass loading of active materials and enhancing energy density, even within a limited footprint area. Nonetheless, the weak adhesion between printed layers engenders consequential intrinsic interfacial hurdles that may impair structural and mechanical robustness. Moreover, this suboptimal adhesion may engender augmented interfacial resistance, thereby instigating amplified polarization, especially for the fast charging/discharging process at a high current density, attenuated electrode conductivity, and hastened electrochemical performance deterioration. To eliminate the interlayer resistance of printed electrodes by layer‐by‐layer stacking, Zhang et al.^[^ ^85^ ^]^ prepared a tempo oxidized cellulose nanofibril (TCNF) mediated conductive functional ink with the self‐healing capability for supercapacitors (Figure 5d). The rapid autonomous self‐healing property of the ink promoted interlayer coalescence instantly after each layer stacking through multiple hydrogen bonds among TCNF‐TCNF, TCNF‐SWCNT, and SWCNT‐SWCNT, resulting in the elimination of interfacial resistance and contributing to efficient active material utilization. As a result, the printed electrode delivered a best‐in‐class electrical double layer (EDL) areal capacitance of 27.1 F cm^−2^ at an extremely high mass loading of 134 mg cm^−2^ and a state‐of‐the‐art energy density of 1.26 mWh cm^−2^ at 24.7 mW cm^−2^. In addition, Zhang et al.^[^ ^52^ ^]^ reported leveraging the utility of a gelation, rapid assembly of active materials into a cellular network with desired rheological behaviors for stable 3D printing at enhanced concentrations (8–20 times) (Figure 5e). The rapid ion and electron transport and the establishment of a self‐healing mechanism enabled by hydrogen bonds of GO and PVA make it possible to eliminate interfacial resistance. As shown in Figure 5f,g, the printed electrode delivers an impressive areal capacitance of 32.2 mg cm^−2^ with a loading density of 2.9 F cm^−2^ and shows an excellent energy density of 0.18 mWh cm^−2^ for supercapacitors.

#### 4.1.3 Improving the Content of Active Materials

**Figure 6:**
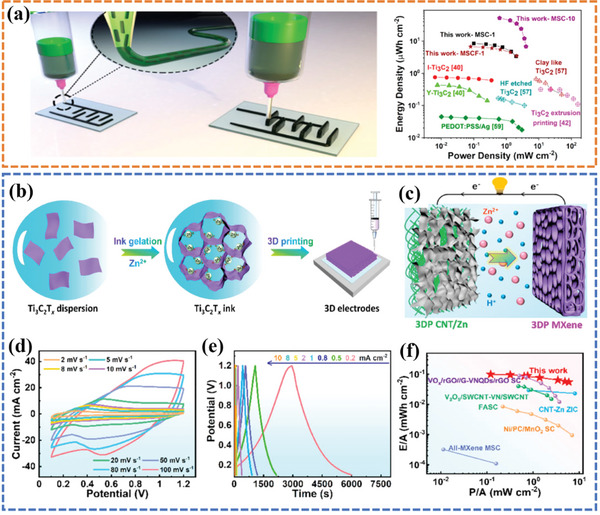
a) Schematic drawing demonstrating the 3D printing of supercapacitors with high power density. Reproduced with permission.^[^ ^61^ ^]^ Copyright 2020, American Chemical Society. b) The 3D printing process of a MXene‐based ink and c) the configuration of an all 3D‐printed ZIC. d) The CV curves e) charging–discharging curves of the ZIC. f) The Ragone plot of assembled ZIC. Reproduced with permission.^[^ ^90^ ^]^ Copyright 2021, American Chemical Society.

Minimizing the quantity of inactive constituents is a feasible and uncomplicated approach to augment the content of active materials. Additionally, this technique is advantageous in mitigating the risk of potential side reactions, such as the breakdown of PVDF during high‐voltage charging, which can impair the electrochemical efficacy of EESDs. Unfortunately, in most conventional inks, it is essential to use a certain content of polymers as rheology modifiers or conductive additives to improve the electronic conductivity, but this accounts for a large proportion of the weight (even in excess of 90%) without contributing any real capacity. By fine‐tuning the concentration of electroactive 2D materials (such as MXene,^[^ ^86^, ^87^ ^]^ graphene,^[^ ^88^ ^]^ etc.), it is possible to regulate their viscoelastic properties, thereby creating novel prospects for the development of 3D printable inks that contain a high proportion of active material.^[^ ^63^, ^89^ ^]^ Majid et al.^[^ ^61^ ^]^ designed a highly concentrated, water‐based, and additive‐free MXene dispersion as a printable ink, showing desirable rheological properties for extrusion‐based printing under room temperature (**Figure** **6**a). The printed structure was mechanically robust in a layer‐by‐layer fashion over a thickness of several millimeters without collapsing, delivering a maximum capacitance of 1035 mF cm^−2^ at 2 mVs^−1^. At the device dimension, the superior conductivity of MXene is exploited without the need for current collectors, further increasing the energy density.^[^ ^60^ ^]^ To achieve high energy density/power density aqueous Zn‐ion hybrid capacitors (ZICs), Sun et al.^[^ ^90^ ^]^ used a 3D printing technology to fabricate a Ti_3_C_2_ MXene cathode for ZICs. The highly concentrated, additive‐free MXene ink exhibited a desirable rheological property derived from a rapid gelling process employing a trace number of divalent cations (Figure 6b). The printed Ti_3_C_2_ MXene cathode exhibited exceptional carrier transport ability, complete electrolyte infiltration, and ample porosity. The ZIC was fabricated by a 3D‐printed MXene cathode and a 3D‐printed Zn anode (Figure 6c). The conspicuous redox of CV curves documented the pseudocapacitive nature of thus‐constructed 3DP ZICs (Figure 6d). The areal capacitance reached 244.6 mF cm^−2^ under a high current density of 10 mA cm^−2^, indicating an excellent rate capability (Figure 6e). Meanwhile, as demonstrated by the Ragone plot in Figure 6f, the device delivered an ultrahigh energy density (0.10 mWh cm^−2^) and power density (5.90 mW cm^−2^), respectively.

### 4.2 High Power Density EESDs

The incorporation of 3D printing technology into the production of high‐mass‐loading electrodes holds promise for enhancing the ion transport capability of 3D electrodes via intricately crafted macro‐pore architectures.^[^ ^26^, ^91^ ^]^ Yet, there is an increasing challenge to balance high areal capacity with high power density arising as the thickness of the 3D electrode. The pronounced extension of the electron conduction and ion transport pathways impairs their rate performance and ultimately undermines the power density of EESDs. Hence, further research and development are necessary to optimize the electrochemical performance of 3D‐printed electrodes, particularly with regard to power density at high mass loading.

#### 4.2.1 Surface/Interface Engineering for Enhanced Electronic Conductivity

##### Element Doping

**Figure 7:**
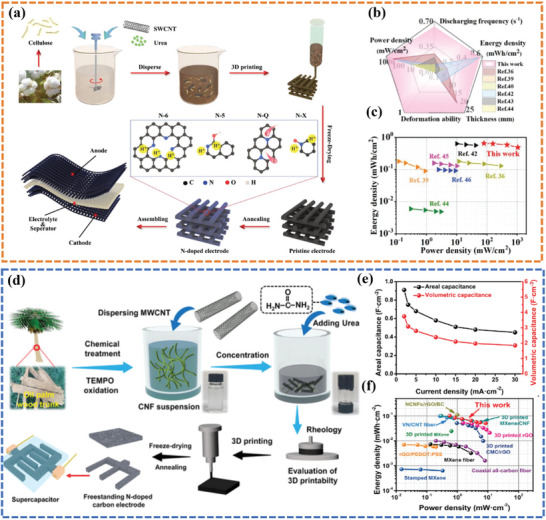
a) A diagram illustrating the overall printing scheme, doping engineering, and structure of an assembled supercapacitor. b) The radar map and c) Ragone plot of this compared with reported symmetric supercapacitors. Reproduced with permission.^[^ ^99^ ^]^ Copyright 2021, Wiley‐VCH. d) A schematic diagram illustrating the fabrication of a quasi‐solid‐state symmetrical supercapacitor using two 3D‐printed N‐doped carbon electrodes. e) The capacitance at different current densities. f) The Ragone plot of the 3D‐printed QSSC device compared with another reported device. Reproduced with permission.^[^ ^100^ ^]^ Copyright 2023, Wiley‐VCH.

Traditionally, one of the most straightforward ways to improve the electrical conductivity of the printed electrodes is to formulate highly conductive additives directly into the inks, such as graphene,^[^ ^31^, ^42^, ^48^, ^92^ ^]^ CNTs,^[^ ^52^, ^85^, ^93^ ^]^ acetylene black ^[^ ^94^, ^95^ ^]^ and Ag nanowires.^[^ ^96^ ^]^ Recently, heterogeneous elemental doping has been considered as another effective strategy to enhance the electrical conductivity of 3D‐printed electrodes by redistributing the charge on the surface of the elements.^[^ ^97^, ^98^ ^]^ Zhang et al.^[^ ^99^ ^]^ reported that N‐doping engineering was utilized to regulate the physical properties and the surface chemistry of 3D‐printed electrodes to encourage high capacity and fast interfacial reaction kinetics, thus addressing the ongoing challenge of performance trade‐off between power performance and energy density at high mass loading (**Figure** **7**a). Remarkably, a 3D‐printed 12.2 mm thick electrode with an exceptionally high loading of 85.1 mg cm^−2^ was capable of flash‐charging in 3.6 s while maintaining 78.1% capacitance, which was comparable to a thin‐film electrode (Figure 7b). The assembled symmetric supercapacitor delivered a maximum power density of 1039.8 mW cm^−2^ at a high energy density of 0.49 mWh cm^−2^ (Figure 7c). Mei et al.^[^ ^100^ ^]^ prepared a hybrid ink consisting of CNFs and CNTs with urea acting as a doping agent (Figure 7d). Notably, the decomposition of urea during the calcination process induced in situ N‐doping, resulting in the formation of structural defects, exceptionally high conductivity, and outstanding wettability in the 3D‐printed electrode. As exhibited in Figure 7d‐f, the thus‐fabricated quasi‐solid‐state symmetric supercapacitor achieved a high areal/volumetric capacitance of 0.91 F cm^−2^/3.74 F cm^−3^ at 2 mA cm^−2^ and an exceptional power density of 8.44 mW cm^−2^ at 0.10 mWh cm^−2^.

##### Surface Modification

**Figure 8:**
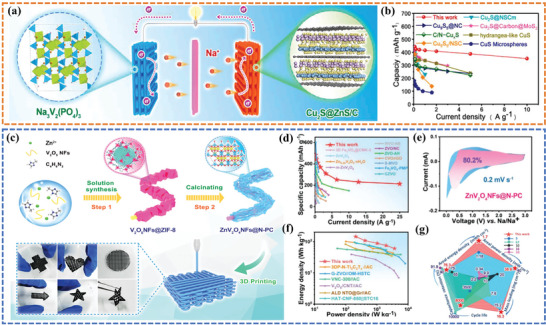
a) Schematic illustration of Cu_2_S@ZnS/C//NVP 3D‐ printed SIBs device. b) Rate capability comparison of Cu_2_S@ZnS/C and reported CuS‐based anode materials for SIBs. Reproduced with permission.^[^ ^101^ ^]^ Copyright 2022, Elsevier. c) Schematic strategy of 3D‐printed ZnV_2_O_4_NFs@N‐PC electrode. d) The capacitances under various current densities. e) Capacitive contribution at a scan rate of 0.2 mV s^−1^. f) The Ragone plot and g) Radar map compared with previous works. Reproduced with permission.^[^ ^102^ ^]^ Copyright 2022, Wiley‐VCH.

Apart from the element doping strategy to improve the electrical conductivity of 3D‐printed electrodes, surface modification offers another alternative, including core‐shell structure, heterojunction interface, surface coating of active material, and so on. Wen et al.^[^ ^101^ ^]^ prepared a heterostructured nanohybrids with ZnS and carbon coating Cu_2_S nanoplates (Cu_2_S@ZnS/C) electrode for sodium‐ion batteries (**Figure** **8**a). The distinctive heterostructures with carbon decorating of Cu_2_S@ZnS/C electrode remarkably accelerated electron conduction and ionic diffusion kinetics while guaranteeing the structural integrity of sodium ion storage. As shown in Figure 8b, the 3D‐printed sodium‐ion batteries with 3D‐printed Cu_2_S@ZnS/C anode and 3D‐printed Na_3_V_2_(PO_4_)_3_ cathode delivered a good rate performance with a reversible capacity of 352 mAh g^−1^ maintaining at 10 A g^−1^. Wen et al.^[^ ^102^ ^]^ also fabricated a 1D core‐shell structure with N‐doped porous carbon encapsulating ZnV_2_O_4_ nanofibers (ZnV_2_O_4_NFs@N‐PC) for sodium‐ion hybrid capacitors (Figure 8c). The rate performance of ZnV_2_O_4_NFs@N‐PC electrode was displayed in Figure 8d, where the electrode retained a high capacity of 213.2 mAh g^−1^ even at an ultrahigh current density of 25 A g^−1^, demonstrating the brilliant rate capability. Based on the CV curve displayed in Figure 8e, the capacitive contribution of the ZnV_2_O_4_NFs@N‐PC anode was determined to occupy a remarkable 80.2% toward the overall capacity at 0.2 mV s^−1^, which serves as compelling evidence for the exceptional pseudocapacitive Na storage predominance and ultrafast kinetics, thereby enabling high‐rate capability. Benefitting from the advantages of the hierarchical porous framework of 3D‐printed ZnV_2_O_4_NFs@N‐PC anode and 3D‐printed active carbon cathode, the 3D‐printed sodium‐ion hybrid capacitors can deliver high areal energy of 1.67 mWh cm^−2^ and power density of 38.96 mW cm^−2^ at a high mass loading of up to 16.25 mg cm^−2^ (Figure 8f,g).

#### 4.2.2 Hierarchical Pore Structure Design Promoting Fast Ion Diffusion Kinetics

**Figure 9:**
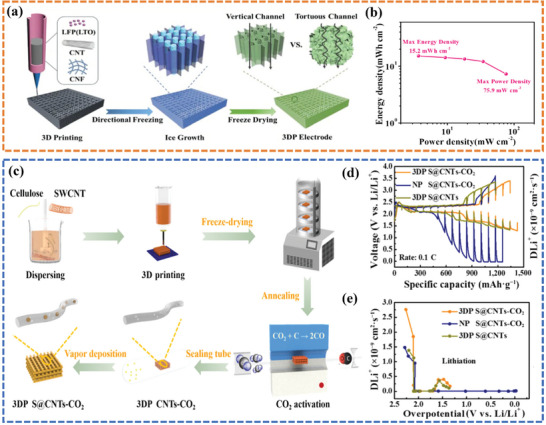
a) Schematic illustration of 3D‐printed flexible LIBs electrode. b) The Ragone plot of a printed 8‐layer full cell. Reproduced with permission.^[^ ^108^ ^]^ Copyright 2022, Wiley‐VCH. c) A schematic illustration of the fabrication process of the 3D‐printed S@CNTs‐CO_2_ electrode. d) GITT test of 3DP S@CNTs‐CO_2_ compared with NP S@CNTs‐CO_2_ and 3DP S@CNTs electrodes. e) Diffusion coefficient of Li^+^ during lithiation process. Reproduced with permission.^[^ ^109^ ^]^ Copyright 2022, Springer Nature Ltd.

The precise engineering of pore architecture in printed electrodes represents a well‐established approach for augmenting ion diffusion kinetics, thereby significantly boosting the power density of EESDs.^[^ ^103^ ^]^ By leveraging the inherent macropores generated during 3D printing, advanced hierarchical pore structures can be achieved through in situ etching,^[^ ^104^, ^105^ ^]^ freeze drying,^[^ ^106^, ^107^ ^]^ or supercritical drying,^[^ ^31^ ^]^ which can effectively impart superior ion transport properties to the printed electrodes. Zhang et al.^[^ ^108^ ^]^ proposed a directional freezing‐assisted 3D printing strategy to fabricate flexible, compressible, and ultra‐high energy/power density LIBs (**Figure** **9**a). The directional freezing‐induced vertical channels could function as fast ion diffusion pathways, effectively overcoming the lagging ion transport limitation of 3D‐printed flexible electrodes with increased mass loading. As expected, the 3D‐printed LIB delivered a record high energy /power density of 15.2 mWh cm^−2^/75.9 mW cm^−2^ (Figure 9b). Later, Zhang et al.^[^ ^109^ ^]^ reported a 3D single‐walled carbon nanotubes/S composite thick electrode (3DPS@CNTs‐CO_2_) with hierarchical pore structure for lithium‐sulfur batteries (LSBs) (Figure 9c). The integration of a 3D hierarchical pore structure with regularly arranged nanopores not only enables the achievement of elevated S mass loading and efficient S encapsulation within the tubes but also enhances the utilization and reaction activity of S while exhibiting outstanding electronic conductivity. These characteristics, in turn, greatly enhance ion diffusion kinetics even at high mass loading. The 3DP S@CNTs‐CO_2_ electrode showed a small overpotential and larger Li^+^ diffusion coefficients (Figure 9d,e), validating the positive effect of CO_2_‐activated CNTs and 3D‐printed electrode architecture design in facilitating the reaction kinetics. Due to these merits, the polarization of the 3DP S@CNTs‐CO_2_ electrode was greatly decreased, and the reversible charge–discharge capacities were held to 3.14/3.11 mAh cm^−2^, further confirming the favorable effect of the 3D printing architecture design in promoting ion diffusion kinetics.

### 4.3 High Volumetric Energy Density EESDs

The optimized 3D‐printed EESDs have the innate ability to effortlessly surpass previous areal energy density records by using layer‐by‐layer stacking, thereby illustrating the seamless synergy between advanced craftsmanship and cutting‐edge technology. The remarkable advancements in portable, wearable, miniature electronic devices and long‐range electric vehicles witnessed in recent decades have made volumetric energy density a crucial and dependable metric for assessing the efficacy of high‐performance EESDs. Notwithstanding, the enhancement of performance in 3D‐printed electrodes with high mass loading requires the presence of copious ion transport pathways and optimal active material utilization, which have perpetually been pivotal factors. The preparation of thick electrodes frequently results in a regrettable compromise between density and porosity, leading to a less‐than‐ideal outcome. Most 3D‐printed electrodes with low packing density (< 0.1 g cm^−3^) fail to meet the expectation of high volumetric energy density for practical applications. How to maintain favorable electrochemical performance based on obtaining high mass loading and compact 3D‐printed electrodes is the premise for realizing the double leap in the areal energy density and volumetric energy density for high‐performance EESDs.

#### 4.3.1 Mechanical Compression

**Figure 10:**
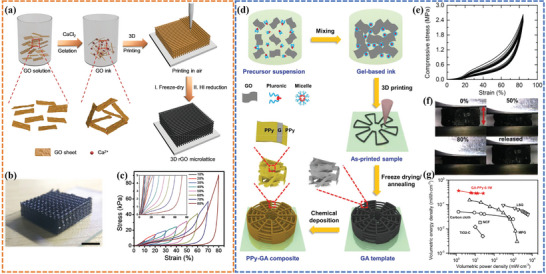
a) Schematic illustration of the fabrication of the 3D‐printed graphene aerogel microlattices (GAMs). b) Images of as‐printed woodpile structure. c) The stress curves as a function of compress strain for printed GO microlattice. Reproduced with permission.^[^ ^65^ ^]^ Copyright 2018, Wiley‐VCH. d) Schematic diagram showing the fabrication process of 3D hierarchical PPy–GA composites. e) Compressive stress–strain curve of PPy‐GA microlattice. f) Optical images of 0–80% strain cycles of a PPy–GA microlattice. g) Volumetric energy and power density of PPy–GA in a Ragone plot. Reproduced with permission.^[^ ^111^ ^]^ Copyright 2018, Wiley‐VCH.

The mechanical compression of 3D‐printed electrodes is a straightforward method for enhancing the packing density of the electrodes. However, this approach comes at the expense of capacity loss, as the irreparable structural damage and deterioration of ion transfer kinetics, resulting from the distortion and blockage of the internal pore architecture, are a direct consequence.^[^ ^110^ ^]^ It is worth noting that the compression of electrodes to enhance their volumetric capacity is at odds with the manufacturing principles of 3D‐printed thick electrodes, which strive to optimize the use of height space. Nonetheless, in most cases, the increase in volumetric energy density achieved by compression is an additional benefit of implementing a compressible electrode. Gao et al.^[^ ^65^ ^]^ have successfully demonstrated a facile ion‐induced gelation technique to prepare 3D printed graphene aerogel microlattices (GAMs) by adding traces of Ca^2+^ ions as gelators to a printable ink with proper rheological behaviors (**Figure** **10**a). The 3D‐printed GAMs showed excellent mechanical properties with exceptional compressive stress of ≈90 KPa under a high strain amplitude of 80% (Figure 10b,c). The supercapacitors with GAMs as electrodes exhibited a high volumetric capacitance of 85.4 F cm^−3^ at 0.4 mA cm^−3^. Zhu et al.^[^ ^111^ ^]^ designed a concentrated, aqueous‐based GO suspension with (NH_4_)_2_CO_3_ as cross‐linker and hydroxypropyl methylcellulose (HPMC)/poly(ethylene oxide) (PEO)‐poly(propylene oxide) (PPO)‐PEO (pluronic F127) as viscosifier to prepare the printable ink (Figure 10d). Poly‐pyrrole (PPy) was then uniformly chemically deposited and adhered to the 3D‐printed graphene aerogel (GA) scaffolds, which not only significantly increased the gravimetric capacitance (395 F g^−1^), but also improved the compressive strength (2.4 MPa) (Figure 10e and f). The symmetric supercapacitor delivered a high volumetric capacitance of 3.5 F cm^−3^ (at 1 mA cm^−3^) and a maximum volumetric energy density of 0.4 mWh cm^−3^ at the power density of 1.2 mW cm^−3^ (Figure 10g). However, the aforementioned work has not extensively investigated the retention of electrochemical performance of 3D‐printed electrodes or devices when subjected to mechanical compression.

**Figure 11:**
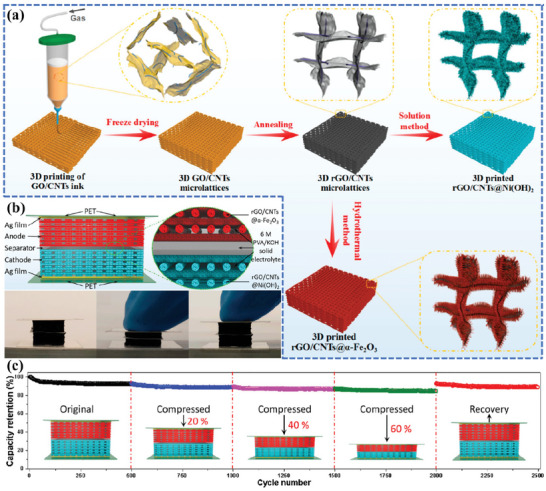
a) Fabrication procedures and schematics of the 3D‐printed Ni−Fe battery. b) Schematic diagram of a 3D‐printed compressible QSS‐NFB device and real‐time photos of the device showing the compressing and recovering process. c) Cycling performance at different compression states, current density: 200 mA cm^−2^. Reproduced with permission.^[^ ^112^ ^]^ Copyright 2020, American Chemical Society.

Yang et al.^[^ ^112^ ^]^ reported a 3D‐printed quasi‐solid‐state Ni‐Fe battery (QSS‐NDB) with outstanding compressibility, ultra‐high volumetric energy density, and excellent cycling performance. The highly conductive and porous rGO/CNT hybrid aerogel was used as a scaffold to in‐suit grow ultrathin Ni(OH)_2_ nanosheet array cathode and holey α‐Fe_2_O_3_ nanorod array anode by solvothermal method (**Figure** **11**a). The 3D‐printed rGO/CNT@Ni(OH)_2_ and rGO/CNT@α‐Fe_2_O_3_ microlattices retained fine compression tolerance at a high strain of 60%, showing that the incorporated active materials could act as buffers and improve the mechanical properties during compression (Figure 11b). Impressively, the 3D‐printed QSS‐NDB devices delivered a high volumetric energy density (28.1 mWh cm^−3^) at a power density of 10.6 mW cm^−3^ and were able to hold 84.5% of their initial capacity after being subjected to different strains over 2000 cycles, demonstrating exceptional electrochemical stability (Figure 11c).

#### 4.3.2 Capillary Shrinkage Densification

**Figure 12:**
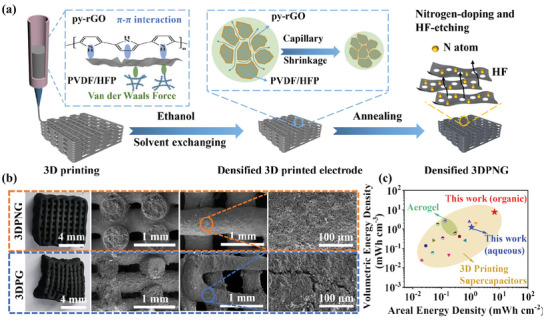
a) Schematic illustration of the fabrication process of the densified 3D‐printed nitrogen‐doped electrode (3DPNG). b) Digital photos and SEM images of 16‐layer densified 3DPNG and densified 3DPG electrode. c) The areal and volumetric energy density in aqueous and organic electrolytes compared with representative literature. Reproduced with permission.^[^ ^104^ ^]^ Copyright 2022, Wiley‐VCH.

Capillary densification presents an additional adaptable approach for augmenting the packing density of electrodes by utilizing the internal capillary forces on the pore walls, which instigate potent shrinkage brought about by the capillary evaporation of a solvent.^[^ ^113^ ^]^ This tactic has already accomplished noteworthy triumph in the production of compact aerogel electrodes intended for EESDs with elevated volumetric energy density.^[^ ^114^, ^115^ ^]^ Zhang et al.^[^ ^104^ ^]^ proposed a pioneering 3D printing technique utilizing a surface‐adaptive capillarity approach to achieve densification, which elevated the performance of a 3D‐printed supercapacitor to an unprecedented level with regard to areal and volumetric energy densities (**Figure** **12**a). With the aid of pyrrole surface‐modified reduced graphene oxide (py‐rGO), the capillary shrinkage of 3D‐printed electrodes was properly manipulated, and the structural integrity of a densified printed electrode was well preserved (Figure 12b). The 16‐layer intensely densified 3D‐printed electrode delivered ultra‐high areal and volumetric capacitances of 16.4 F cm^−2^ and 36.4 F cm^−3^, respectively, under extremely high areal mass loading (145 mg cm^−2^) and packing density (322.2 mg cm^−3^). In addition, in situ N‐doping and HF‐etching induced hierarchical micro‐meso porosity were simultaneously achieved during post‐carbonization, promoting enhanced EDL capacitance and fast ion diffusion. In aqueous/organic electrolyte, this amounts to impressive areal/volumetric energy densities of 1.12/7.01 mWh cm^−2^ and 1.24/7.79 mWh cm^−3^ at 2 mA cm^−2^ for symmetrical EDL device performance, which is superior to all the reported 3D‐printed supercapacitors and even many thick aerogel supercapacitors (Figure 12c).

### 4.4 Excellent Mechanical Flexibility EESDs

Flexible and wearable electronics, such as smart wristbands (Apple Watch), smart glasses (Google Glass), and fitness trackers (Fitbit), have attracted widespread interest in recent years and are already deeply ingrained in everyday life.^[^ ^116^, ^117^, ^118^, ^119^ ^]^ As an indispensable functional component of wearable devices, energy storage devices are facing expanding performance requirements due to the ever‐increasing power consumption of multi‐functional electronics. Commercially energy storage devices, especially lithium‐ion batteries and supercapacitors are typically bulky and rigid, which does not match the stringent requirements for wearable energy storage devices (WESDs) in terms of portability and flexibility. In recent decades, various fabrication technologies, such as photolithography, e‐beam and ion‐beam lithography, and templating printing methods, have been widely used to fabricate WESDs^[^ ^120^ ^]^ with decent electrochemical performance and mechanical flexibility. Stable energy output under arbitrary mechanical deformation is desperately required for ideal flexible WESDs with uncompromising electrochemical performance (including energy density, power density, cycling performance, etc.), safety performance, and mechanical properties. To relieve the internal stress during deformation and thus improve the mechanical properties of WESDs, solutions are generally proposed from the perspective of formulation optimization and geometric patterning. DIW 3D printing can simultaneously share the benefits of multi‐material printing capability and high design versatility of complex structures, providing a powerful tool for the fabrication of flexible WESDs.

#### 4.4.1 Coupling of Functional Components

**Figure 13:**
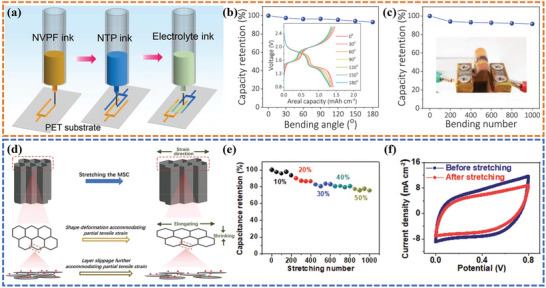
a) Schematic of the aqueous NVPF and NTP microelectrode inks and ionogel electrolyte ink for 3D printing NVPF||IE||NTP NIMB/EC. b) Capacity retention as a function of bending angles. c) Capacity retention as a function of repeated bending number. Reproduced with permission.^[^ ^128^ ^]^ Copyright 2022, Wiley‐VCH. d) Schematic illustration of the slippage of the internal structure of the 3D‐printed electrodes under deformation. e) Capacitance retention as a function of stretching/releasing cycle number under different strains. f) CV curves before and after 1000 stretching cycles. Reproduced with permission.^[^ ^130^ ^]^ Copyright 2020, Wiley‐VCH.

The utilization of carbon‐based materials, such as graphene, carbon nanotubes, or carbon fibers, has become a versatile and widely adopted strategy for the preparation of flexible electrodes.^[^ ^121^, ^122^, ^123^, ^124^ ^]^ As common components of inks, they are also extensively employed in the fabrication of flexible 3D‐printed EESDs.^[^ ^93^, ^125^, ^126^, ^127^ ^]^ Wu et al.^[^ ^128^ ^]^ prepared 3D printed microelectrode inks consisting of Na_3_V_2_(PO_4_)_2_O_2_F (NVPF, cathode) or carbon‐coated NaTi_2_(PO_4_)_3_ (NTP, anode), 2D exfoliated graphene (EG) and 1D carbon nanotubes (CNTs) for the construction of flexible sodium‐ion microbatteries (NIMBs) with a planar interdigital configuration, and NaBF_4_‐based ionogel was used as electrolyte (denoted as NaBF_4_‐IE) (**Figure** **13**a). A 2‐layer NVPF||NaBF4||NTP NIMB/EC with a microelectrode thickness of ≈300 µm was assembled to measure the electrochemical performance in the bent state. The superior capacity retention of 93% was achieved at a high 180° bending angle (Figure 13b) and 91% of the initial capacity was retained even after repeated bending cycles over 1000 times (Figure 13c). In a typical ink formulation, binders and additives are also important components that play a pivotal role in tuning the rheological properties and electrical conductivity. They have also been applied to ameliorate the mechanical properties of printed electrodes. Several polymeric binders, such as phenol formaldehyde (PF),^[^ ^129^ ^]^ and pluronic F127,^[^ ^111^ ^]^ have been used to disperse the strain to yield satisfactory mechanical properties. Besides, the additives can serve as surface tension modifiers to optimize appropriate mechanical flexibility. Li et al.^[^ ^130^ ^]^ fabricated an intrinsically stretchable micro‐supercapacitor by optimizing the selection of printable materials, consisting of MXene nanosheets, Ag nanowires, MnO_2_ nanowires, and fullerene (C60). The C60 was incorporated into the cell wall and acted as a lubricant to allow adjacent MXene nanosheets to slippage during the partial structural deformation (Figure 13d). As a result, the areal capacitance of the printed electrode decreased by less than 20% under up to 50% tensile strain and retained a high capacitance retention rate of 75% after 1000 stretch/release cycles (Figure 13e). The CV curves after 1000 cycles showed no noticeable change in a rectangular shape, indicating excellent resilience to stretching and bending (Figure 13f). Furthermore, the ink's exceptional self‐healing capability, attributed to the interactions among its functional components, including dynamic hydrogen bonding and van der Waals forces, also plays a pivotal role in facilitating the favorable mechanical deformation and subsequent recovery of the flexible electrodes.

#### 4.4.2 Geometric Patterning Design

**Figure 14:**
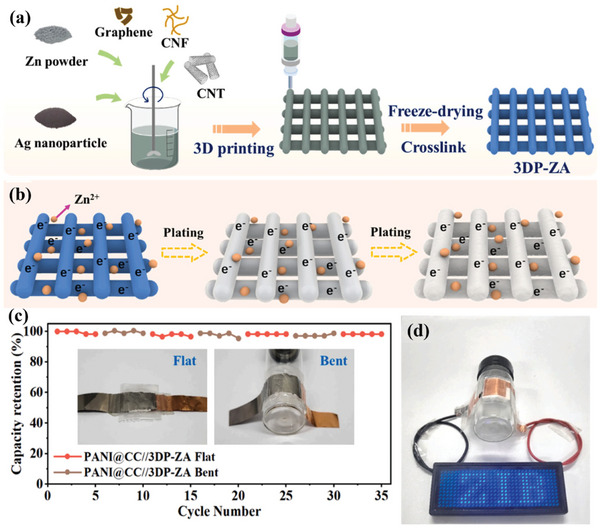
a) The schematic illustration of the preparation process of the 3DP‐ZA anode. b) The illustration of the deposition process of 3DP‐ZA anode. c) The capacity of PANI@CC//3DP‐ZA full cell under continuous deformation states, inset: the photos of the flat and bent device. d) Three PANI@CC//3DP‐ZA full cells connected in series under bent state light the LED board. Reproduced with permission.^[^ ^131^ ^]^ Copyright 2022, Elsevier.

Geometric patterning design represents another alternative strategy that can effectively buffer the strain force under deformation. As of now, a multitude of 3D‐printed flexible electrodes have been fabricated based on structural engineering principles, with the goal of achieving enhanced mechanical flexibility. Zhang et al.^[^ ^131^ ^]^ proposed a novel 3D‐printed Ag‐anchored Zn anode (3DP‐ZA) for flexible dendrite‐free ZIBs (**Figure** **14**a). By introducing a 3D‐printed structure with customized macropores, which provide sufficient buffer space for stress relaxation and accommodate volume changes during plating/stripping, the 3DP‐ZA flexible electrode achieved dendrite‐free behavior and displayed excellent structural integrity (Figure 14b). A typical full cell was assembled employing 3DP‐ZA as the anode and PANI‐coated carbon cloth (PANI@CC) as the cathode (denoted as PANI@CC//3DP‐ZA), and its electrochemical performance was further investigated under different distortion conditions at 1.0 A g^−1^. It was found that there was no apparent capacity decay over 35 cycles when the full cell was wrapped around a 3 cm diameter glass bottle (Figure 14c). Three full cells serially connected in a bent state could readily power a LED board (3.7 V), demonstrating excellent mechanical flexibility and long‐term electrochemical stability (Figure 14d).

**Figure 15:**
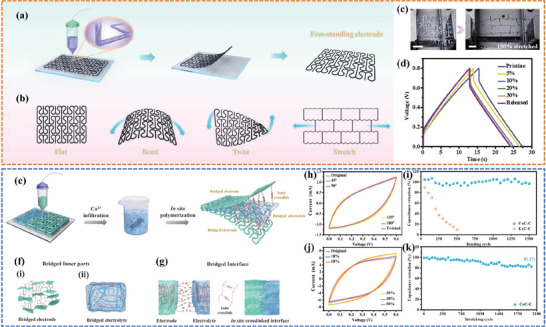
a) Schematic illustration of the fabrication process of the conducting polymer electrode. b) Schematic illustration of the 3D‐printed electrodes under different deformation conditions. c) S‐hinged structure electrodes in the maximum stretch strain of 150%. d) GCD curves of the stretchable supercapacitor under different stretching strains. Reproduced with permission.^[^ ^134^ ^]^ Copyright 2022, Royal Society of Chemistry. e) Illustration of the structure and manufacturing process of the supercapacitor. f) Illustration reflecting the structure of supercapacitors with multi‐level bridged configurations. g) Illustration of the formation mechanism of the bridged interface based on ionic bonding. h) CV curves of supercapacitors obtained under different bending states. i) Capacitance retention as a function of bending cycles. j) CV curves of supercapacitors with various tensile strains. k) Cycling performance of supercapacitor over 2000 stretching cycles. Reproduced with permission.^[^ ^66^ ^]^ Copyright 2022, Royal Society of Chemistry.

The unique mechanical properties of structures with negative Poisson's ratio (NPR), which contract laterally under compression and expand laterally under tension, make them ideal for 3D‐printed stretchable electrodes. These properties provide excellent fracture toughness, enhanced shear modulus, and outstanding shape adaptability, which are essential for creating durable and flexible electrodes.^[^ ^132^, ^133^ ^]^ Huang et al.^[^ ^134^ ^]^ designed a free‐standing stretchable and flexible electrode with an NPR (S‐hinged) structure based on a poly(3,4‐ethylene dioxythiophene): polystyrene sulfonate/CNT (PEDOT: PPS/CNT) ink (**Figure** **15**a,b). The 3D‐printed electrode with an optimized arc‐shaped NPR structure exhibited favorable flexibility (bent to 180°) and extreme stretchability (maximum elongation 150%) (Figure 15c). The galvanostatic charge‐discharge (GCD) curves were measured for a quasi‐solid‐state symmetric supercapacitor under different stretching strains, and it was found that the stretchable supercapacitor exhibited stable electrochemical performance with little variation in capacitance (Figure 15d). In a follow‐on work,^[^ ^66^ ^]^ the authors reported a versatile manufacturing strategy to prepare integrated deformable supercapacitors with multi‐level bridged configurations. The alginate chains were in‐suit polymerized to introduce multi‐level tight connections in both the electrolyte and electrode, which not only improves the robustness and tensile strength of each component but also strengthens the interfacial bonding for better ionic contact (Figure 15e–g). As a result, high efficiency of interfacial charge transfer and stable electrochemical performance under harsh deformation conditions have been successfully achieved. The CV curves of the assembled supercapacitor basically overlapped as the bending angle was increased from 0° to 180° (Figure 15h) and the supercapacitor maintained a high capacity retention rate of ≈97% after 1500 bending cycles (Figure 15i). Furthermore, benefiting from the S‐hinged NPR structure, the device retained excellent capacitance performance even when stretched to a maximum strain of 50% (Figure 15j), and kept 83.2% of its initial capacitance after 2000 repeated stretch/release cycles (Figure 15k).

**Figure 16:**
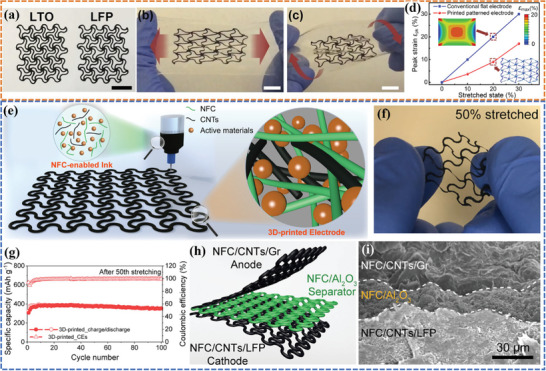
a) Optical photos of the as‐fabricated stretchable LTO and LFP electrodes. The deformed shape of the sandwiched electrodes under b) stretching and c) twisting. d) The comparison of peak strain ε_pk_ in the conventional straight electrode and the suitably patterned electrode at different stretched states.^[^ ^135^ ^]^ Reproduced with permission. Copyright 2020, Elsevier. e) Compositional and morphological features of the ink for 3D printing of the stretchable battery components. f) Photos of the 3D printed electrode under stretched states along the diagonal direction with a stretch strain of 50%. g) Cycling performance of the 3D‐printed electrode after the 50th stretch‐release cycle. h) Schematics illustrating the sandwich‐structured stretchable battery. i) Cross‐sectional SEM image of the designed sandwich structure with 3D‐printed electrodes and separator. Reproduced with permission.^[^ ^136^ ^]^ Copyright 2022, Elsevier.

Li et al.^[^ ^135^ ^]^ reported 3D‐printed deformable electrodes that could withstand large in‐plane strain by designing a planar network of serpentine (**Figure** **16**a) based on LFP/LTO:MWCNT:PVDF inks. The electrodes sandwiched between two PDMS membranes could be subject to stretching (Figure 16b) or twisting (Figure 16c) while maintaining structural integrity under deformation, demonstrating excellent structural flexibility. The strain relief mechanisms were validated by finite element analysis (FEA) and tensile tests, which showed that the tensile strain in the printed electrode was kept at a low level compared to the conventional flat electrode (Figure 16d). The same group has also developed aqueous inks using nanofibrillated cellulose (NFC) to fabricate stretchable battery components with a serpentine structure (Figure 16e).^[^ ^136^ ^]^ The strong hydrogen interactions between NFC and CNTs in the design 3D‐printed network enabled exceptional stretchability of 50% along the diagonal direction (Figure 16f) and stable electrochemical performance of the electrodes (Figure 16g). In addition, a serpentine‐structured separator formulated with NFC and Al_2_O_3_ nanoparticles was prepared, and a preliminary demonstration of a sandwich‐structured stretchable battery was fabricated by adopting a layer‐by‐layer printing strategy (Figure 16h). The sandwich structure could be preserved during the preparation process without mixing or collapsing due to the good printability of the inks (Figure 16i). However, while this design showed promise for creating stretchable electrodes, a full stretchable battery was not yet demonstrated. Further research and development are needed to create a complete battery with the necessary stretchability and electrochemical performance.

**Figure 17:**
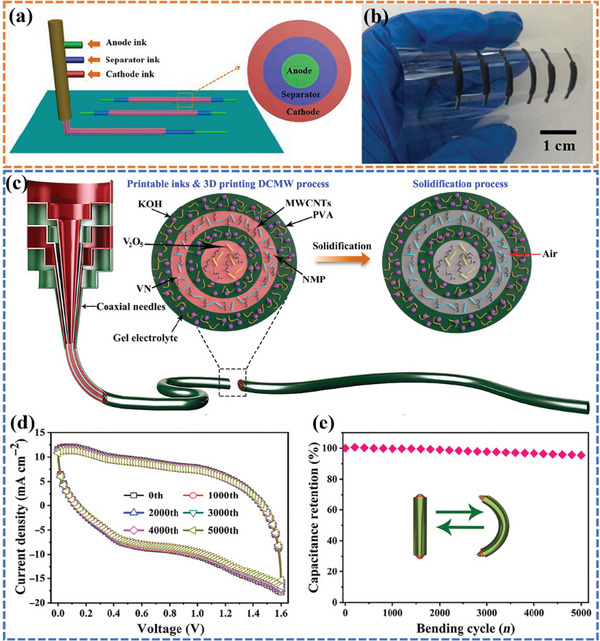
a) Schematic of coaxial 3D‐printing constructing all‐in‐one fibrous LIBs, SIBs, and AZIBs. b) Optical images of the prepared fibrous filaments without encapsulation.^[^ ^138^ ^]^ Reproduced with permission. Copyright 2021, Elsevier. c) 3D printing extrusion process of the printable coaxial FASC device. d) CV curves obtained at the different bending cycles. e) Capacitance retention after 5000 cycles. Reproduced with permission.^[^ ^139^ ^]^ Copyright 2021, American Association for the Advancement of Science.

Fiber‐shaped energy storage devices stand out among the various flexible wearable energy storage devices due to their exceptional flexibility, seamless integration with the traditional textile industry, and unparalleled miniaturization capabilities.^[^ ^137^ ^]^ As a result, they exhibit immense potential for application in smart flexible electronics, thus paving the way for a myriad of innovative and cutting‐edge technological advancements. Ding et al.^[^ ^138^ ^]^ applied a universal coaxial 3D printing strategy to fabricate the all‐in‐one fiber LIBs, sodium‐ion batteries (SIBs), and ZIBs with excellent electrochemical performance in one step (**Figure** **17**a,b). Chen et al.^[^ ^139^ ^]^ proposed a rationally designed 3D printing direct coherent multi‐ink writing (DCMW) technology. An all‐in‐one coaxial fiber‐shaped asymmetric supercapacitor (FASC) device was successfully fabricated by designing the internal structure of the coaxial needles, adjusting the rheological properties and the extrusion rates of the multi‐ink (including V_2_O_5_/MWCNTs ink, gel electrolyte ink, and VN/MWCNTs ink) (Figure 17c). The as‐printed FASC device delivered a superior areal energy/power density (54.3 µWh cm^−2^/801.4 µW cm^−2^) at a high mass loading (16.4 mg cm^−2^) due to the compact coaxial structure. The device managed to keep no substantial deviation of CV curves (Figure 17d) and maintained a high capacitance retention rate of 95.5% over 5000 bending cycles (Figure 17e), showing excellent mechanical flexibility and reliable electrochemical performance under deformation.

**Figure 18:**
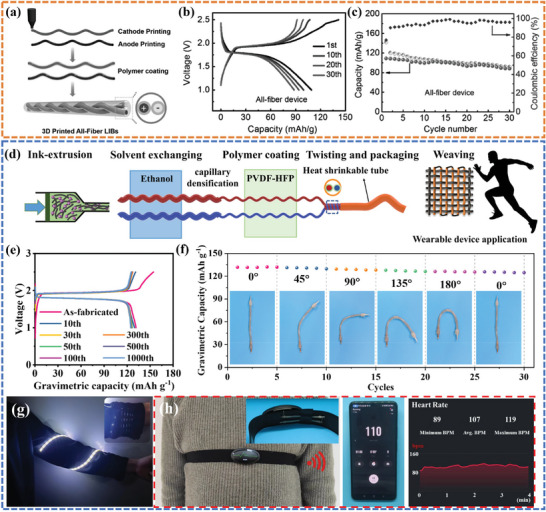
a) Schematic of the design concept and fabrication process of 3D printed all‐fiber flexible LIBs. b) Charge and discharge profiles of the all‐fiber LIB device. c) Cycling stability profiles of the all‐fiber LIB device. Reproduced with permission.^[^ ^46^ ^]^ Copyright 2017, Wiley‐VCH. d) Schematic illustration of the fabrication process of a flexible fiber LIB. e) Cycling performance at 1 C with various bending times. f) Relationship between gravimetric capacity and bending angle. g) LED strips and h) a heart rate chest strap powered by fiber LIB units integrated in series. Reproduced with permission.^[^ ^141^ ^]^ Copyright 2022, Wiley‐VCH.

Compared to the coaxial structure, the twisted structure makes it easier to provide energy storage devices with smaller, lighter, and more flexible features.^[^ ^140^ ^]^ In the pioneering work of Hu et al.,^[^ ^46^ ^]^ they designed and fabricated all‐fiber quasi‐solid‐state LIBs using DIW 3D printing. By coating the surface of fiber electrodes with the gel electrolyte poly(vinylidene fluoride‐co‐hexafluoropropylene) (PVDF‐co‐HFP) and sealing it in a heat shrink tube, a highly integrated all‐fiber LIB structure was successfully fabricated (**Figure** **18**a). The device maintained a high capacity retention of 81% after 30 cycles at 50 mA g^−1^ (Figure 18b,c). However, the electrochemical performance under deformation conditions was not demonstrated. Zhang et al.^[^ ^141^ ^]^ invented an ingenious ink to fabricate flexible, scalable, and high‐mass‐loading fiber LIBs using ink‐extrusion 3D printing technology (Figure 18d). By adopting a densifiable functional ink, the embarrassing dilemma of the low energy density of previously reported fiber LIBs has been effectively addressed. In the prepared inks, py‐rGO not only assembled CNTs and PVDF‐HFP into a sturdy, conductive, and agglomeration‐free 3D network, enhancing the flexibility of the fiber electrodes, but also induced capillary densification. As a result, an ultra‐high active material content of 75% and an extremely high linear mass loading/packing density (1.01 mg cm^−1^ per fiber/782.1 mg cm^−3^) were achieved, which further translated into a high linear/volumetric energy density of 0.26 mWh cm^−1^ per fiber/106.85 mWh cm^−3^. The full cell was able to remain 96.1% of its initial capacity after 1000 repeated bending cycles (Figure 18e) and showed almost no performance degradation under different bending angles (Figure 18f), suggesting exceptional flexible energy storage capability and durability. In applications of various electronic devices, the fiber batteries showed their high practical value in the fields of monitoring/sensing and flexible wearable electronic devices (Figure 18g,h).

In this paper, we systematically discussed the valuable strategies for achieving high‐performance and multifunctional EESDs by classifying the critical performance characteristics, including areal energy density, power density, volumetric energy density, and mechanical flexibility. The corresponding performance and parameter comparisons are listed in **Table** **1**.

**Table 1:** Summary and comparison of the strategies for improving areal energy density, power density, volumetric energy density, and mechanical flexibility.

Parameters	Strategies	Electrode/Device	Type of device	Performance	Reference
Areal energy density	Improve active materials utilization	3DP LTO,3DP LFP	LIBs	4.45 mAh cm^−2^ at 0.14 mA cm^−2^	[83]
		3DP NCM811	LIBs	7.48 mAh cm^−2^ at 16.56 mA cm^−2^	[84]
	Eliminate interlayer resistance	3DP CNF/SWCNT	SCs	27.1 F cm^−2^ at 0.5 A g^−1^	[85]
		3DP PVA/rGO	SCs	2.9 F cm^−2^ at 0.4 A g^−1^	[52]
	Improve active materials content	3DP Mxene	SCs	1035 mF cm^−2^ at 2 mVs^−1^	[61]
		additive‐free MXene cathode	ZICs	244.6 mF cm^–2^ at 10 mA cm^–2^	[90]
Power density	Surface/interface engineering	3DP NC	SCs	1039.8 mW cm^−2^ at 0.49 mWh cm^−2^	[99]
		N‐CNT	SCs	8.44 mW cm^−2^ at 0.10 mWh cm^−2^	[100]
		Cu_2_S@ZnS/C	SIBs	352 mAh g^−1^ at 10 A g^−1^	[101]
		ZnV_2_O_4_NFs@N‐PC	SICs	38.96 mW cm^−2^ at 1.67 mWh cm^−2^	[102]
	Pore structure design	3DP LFP/CNT/CNF	LIBs	75.9 mW cm^–2^ at 15.2 mWh cm^–2^	[108]
		3DPS@CNTs‐CO_2_	LSBs	1.74 mAh cm^−2^ at 1 C	[109]
Volumetric energy density	Mechanical compression	3DP GAMs	SCs	85.4 F cm^−3^ at 0.4 mA cm^−3^	[65]
		3DP GA‐ppy	SCs	3.5 F cm^−3^ (at 1 mA cm^−3^) 0.4 mWh cm^−3^ at 1.2 mW cm^−3^	[111]
		3DP rGO/CNT@Ni(OH)_2_, 3DP rGO/CNT@α‐Fe_2_O_3_	Ni‐Fe	10.6 mW cm^−3^ at 28.1 mWh cm^−3^	[112]
	Capillary shrinkage densification	3DP py‐rGO/PVDF‐HFP	SCs	1.24 mWh cm^−3^	[104]
Mechanical flexibility	Coupling of functional components	3DP NVPF, 3DP NaBF4, 3DP NTP NIMB/EC	SIBs battery	93% capacity retention at 180° bending angle	[128]
		3DP MXene	SCs	75% capacity retention after 1000 stretch/release cycles	[130]
	Geometric patterning design	3DP ZA	ZIBs	no capacity decay after 35 cycles under bending	[131]
		3DP PEDOT: PPS/CNT	SCs	≈97% capacitance retention after 1500 bending cycles	[66]
		3DP NFC/CNTs/Gr3DP NFC/CNTs/LFP	LIBs	no capacity decay at 50% stretch	[136]
		3DP V_2_O_5_/MWCNTs3DP VN/MWCNTs	SCs	95.5% capacitance retention after 5000 bending cycles	[139]
		3DP py‐rGO/CNT/PVDF‐HFP	LIBs	96.1% capacitance retention after 1000 bending cycles	[141]

## 5 Summary and Outlook

DIW is an emerging bottom‐up manufacturing technology that has demonstrated unique advantages in energy storage devices, integrated wearable devices, and microelectronic devices. At present, ink formulas of electrodes/electrolytes and related configurations of practical devices have been well‐established and successfully constructed. DIW technology is anticipated to disrupt the traditional manufacturing paradigm and significantly improve the electrochemical performance of EESDs. Nevertheless, the widespread application of DIW in EESDs in the future is confronted by several formidable challenges, which encompass the meticulous selection of electrode materials, optimal packing density of printed electrodes, ensuring interface stability, improving printing accuracy and speed, as well as enhancing the repeatability and stability:

Electrode material selection and performance optimization. At present, the existing options for DIW 3D printing materials are constrained and fail to fulfill the performance prerequisites of EESDs. Trapped by the irregular size and shape of their powder particles, and the difficulty of nanoparticulation, the applicability of traditional electrode materials in DIW 3D printing is facing great challenges. Consequently, it is imperative to advance the development of novel printable electrode materials, encompassing functional nanocomposites featuring reduced particle sizes and consistent shapes, while simultaneously investigating novel material combinations and synthesis techniques to augment the assortment of DIW 3D printing‐compatible materials. This will facilitate the standardization of materials and establish a comprehensive database of material properties, thereby rendering invaluable contributions to the advancement of high‐performance 3D‐printed energy storage devices. In addition, during the formulation process of electrode ink, it is customary to incorporate large amounts of rheology modifiers and conductive components to uphold the structural integrity and printability of the electrode, which severely reduces the content of active materials, resulting in a decreased overall energy density of the device. Hence, meticulous optimization of interfacial engineering techniques is imperative to maximize the surface modification of electrode materials, thereby enabling the exploration of 3D printable inks containing full active materials, which is an essential prerequisite for the construction of energy storage devices with exceptional energy density.

Improving the packing density of printed electrodes. The implementation of DIW technology allows for the creation of 3D hierarchical pore structure at the macro/micro‐to‐nanoscale level or vertically aligned pores, promoting fast electron/ion transport and effective electrolyte penetration in thick electrodes, ultimately guaranteeing exceptional areal energy density, power density, and rapid charge‐discharge rates in printed devices. However, the existence of a considerable abundance of porous structures in printed electrodes results in diminished electrode density, thereby imposing severe constraints on the enhancement of volumetric energy density. Despite the elucidation of certain strategies aimed at amplifying the volumetric energy density of printed devices, the current state of research concerning the attainment of controlled densification in printed electrodes remains relatively restricted, and the intricate mechanisms governing the process of densification in energy storage systems still lack comprehensive understanding. Hence, it is imperative to embark upon the development of novel strategies, such as interfacial chemistry, plasma etching, and strain engineering, to effectively realize the goal of attaining controlled densification in printed electrodes. Through an in‐depth exploration of the correlation between porosity, electrode density, and electrochemical performance in printed electrodes, coupling with an understanding of the densification mechanisms inherent in diverse energy storage systems, it becomes plausible to simultaneously enhance both the areal energy density and volumetric energy density while upholding high power density.

Enhancing interface stability. EESDs are typically comprised of various constituents such as electrodes, electrolytes, and separators. The interface interaction between these components during the DIW 3D printing process exerts a profound influence on the overall performance of the device. Nonetheless, suboptimal interface connectivity or incompatible responses among materials can result in a decline in device efficacy. Hence, meticulous investigation and enhancement of interface connectivity and compatibility across distinct components are imperative to augment the efficiency of EESDs. By meticulously fine‐tuning printing parameters and judiciously selecting appropriate materials, interface contact between materials can be optimized, while interface engineering techniques can be utilized to mitigate interface reactions and bolster interface stability. In addition, to minimize interface contact issues caused by assembly processes, coaxial or multi‐axis 3D printing techniques can be employed to achieve fully integrated 3D printing of energy storage devices.

Improving printing accuracy and speed. Currently, the accuracy and speed of DIW 3D printing are comparatively subpar, posing challenges in satisfying the demanding prerequisites of EESDs for utmost precision and mass production capabilities. Additionally, the printing process becomes increasingly intricate as it requires precise manipulation of the flow dynamics and intricate interplay among multiple materials. To overcome these challenges, we can elevate the control system of DIW 3D printing processes, integrate innovative print head designs, fine‐tune printing parameters, and refine printing path planning algorithms, ultimately attaining unparalleled levels of precision and efficiency. In particular, incorporating multi‐head printing or parallel printing techniques can enhance concurrent printing operations and boost printing speed, while combining DIW 3D printing technology with advanced manufacturing technologies like robotics can enable automated and streamlined production processes.

Enhancing repeatability and stability. In the realm of DIW 3D printing, the presence of parameter fluctuations and material heterogeneity can give rise to divergent printing outcomes, thereby exerting an adverse influence on the reproducibility and stability of EESDs. To mitigate these challenges, it is imperative to establish standardized DIW 3D printing protocols and implement rigorous quality control measures to ensure consistent parameters and uniform materials, while also incorporating real‐time monitoring, feedback systems, and advanced characterization techniques to assess and optimize printing outcomes, ultimately enhancing the repeatability and stability of EESDs.

In summary, although DIW is a promising method for producing advanced energy storage devices, it is still in its early stages of practical application and requires further development and optimization to achieve widespread commercialization. Indeed, to achieve successful commercialization of DIW in the production of energy storage devices, more attention must be given to optimizing printable universal inks that can provide both good mechanical performance and electrochemical properties. Additionally, the development and optimization of high‐resolution printing equipment are crucial to enable precise and efficient printing of complex structures. Moreover, it is essential to achieve satisfactory production efficiency in large‐scale manufacturing to ensure cost‐effective and reliable production of energy storage devices. Therefore, further research and development are needed to address these challenges and realize the full potential of DIW technology in energy storage applications. As DIW technology continues to be developed and optimized, we can anticipate that the revolutionary device architecture and superior electrochemical performance made possible by this technology will become available in the near future. As research and development efforts continue, we can look forward to exciting advances and breakthroughs in the field of energy storage and beyond.

## Conflict of Interest

The authors declare no conflict of interest.
